# Factors Associated with Cancer Prevention/Risk Reduction Behaviors among Latinos

**DOI:** 10.1007/s40615-023-01895-w

**Published:** 2023-12-20

**Authors:** Susan M. Rawl, Gerardo Maupome, Lilian Golzarri-Arroyo, Erik Parker, Heather A. O’Leary, Manuel R. Espinoza-Gutarra, Robin E. Valenzuela, Caeli Malloy, Laura Haunert, David A. Haggstrom

**Affiliations:** 1 School of Nursing, Indiana University Indianapolis, Indianapolis, IN USA; 2 Indiana University Simon Comprehensive Cancer Center, Indianapolis, IN USA; 3 Richard M. Fairbanks School of Public Health, Indiana University Indianapolis, Indianapolis, IN USA; 4 School of Public Health, Indiana University Bloomington, Bloomington, IN USA; 5 Department of Integrative Medicine, Northeast Ohio Medical University, Rootstown, OH USA; 6 O’Neal Comprehensive Cancer Center, University of Alabama, Birmingham, AL USA; 7 Radius Global Market Research, Mason, OH USA; 8 Center for Health Services Research, Regenstrief Institute, Indianapolis, IN USA; 9 VA HSR&D Center for Health Information and Communication, Richard L. Roudebush Veterans Affairs Medical Center, Indianapolis, IN USA; 10 Division of General Internal Medicine and Geriatrics, Indiana University School of Medicine, Indianapolis, IN USA

**Keywords:** Hispanic/Latino, Population health, Cancer risk perceptions, Cancer prevention behaviors

## Abstract

Improving understanding of behaviors that increase or reduce cancer risk for different Hispanic groups is a public health priority; such knowledge is sparse in new gateway immigration locations such as Indiana. The aims of this study were to: 1) describe cancer beliefs and cancer preventive/risk reduction behaviors (physical activity, tobacco, and alcohol use) among Hispanic adults; 2) examine differences in cancer beliefs and preventive behaviors by country/territory of birth, socioeconomic status, and area of residence (urban vs. rural); and 3) determine predictors of engagement in cancer prevention and risk reduction behaviors in this population. A cross-sectional online survey targeted adult Indiana residents who identified as Latino, Hispanic, or Spanish recruited using Facebook-targeted advertising. Complete survey data from 1520 respondents were analyzed using descriptive, unadjusted, and adjusted models. The majority of respondents believed they were unlikely to get cancer but held many other fatalistic beliefs about cancer. Only 35.6% of respondents had received the HPV vaccine, 37.6% reported they were currently smoking cigarettes, and 64% reported occasional or frequent drinking of alcohol. Respondents spent an average of 3.55 days per week engaged in moderate exercise. Differences were observed by country/territory of birth, income, and education but not by rural residence status. Predictors of cancer risk/risk reduction behaviors were identified. The Hispanic population in Indiana is diverse and effective interventions for cancer prevention should be culturally targeted based on country/territory of birth and individually tailored based on cancer-related beliefs.

## Introduction

The Hispanic/Latino population grew by 23% since 2010 and comprises the largest community of color in the U.S. Numbering more than 62 million, Hispanics represent 19% of the nation’s population [1]. According to the American Cancer Society, cancer is the leading cause of death for Hispanics nationally, accounting for 20% of deaths in 2019 [2] and is the leading cause of deaths for Hispanics in Indiana, accounting for 19% of deaths [3]. Nationally, Hispanics have higher rates of acute lymphoblastic leukemia as well as cervical, stomach, liver, and gallbladder cancers than non-Hispanic whites [4]. Cervical cancer incidence and mortality rates among Hispanic women are 30–40% higher than those of non-Hispanic whites [4]. For Hispanic men, liver and stomach cancer incidence and mortality rates are approaching double and, for women, are more than double those of non-Hispanic whites [4]. Moreover, Hispanics are more likely to be diagnosed with cancer at a later stage. From 2014–2018, only 59% of breast cancers in Hispanic women were diagnosed at an early stage compared to 67% of non-Hispanic white women. Hispanic women are more likely to be diagnosed with larger tumors that are hormone receptor negative, making their breast cancers more difficult to treat [4].

The Hispanic population in Indiana almost doubled between 2000 and 2010 and increased by another 40% to represent 8.2% of the state’s total population in 2020 [5]. The growth in the number of Hispanics in Indiana is expected to continue. Understanding the cancer prevention and risk reduction behaviors and needs of this growing population will help improve health outcomes and reduce cancer risk.

It has been estimated that 42% of all cancer cases and 45% of cancer deaths could be prevented if people adopted healthy behaviors [6]. These behaviors include not smoking, maintaining a healthy weight, staying physically active, eating a healthy diet, and limiting alcohol intake. In addition, regular screening can prevent some cancers or detect them at an early stage when a complete cure is possible. Cancer screening participation in eligible Hispanic populations in Indiana was found to be much lower than other racial/ethnic groups; only 44% of Hispanic women reported being up to date with breast cancer screening, 61% were up to date with cervical cancer screening and 68.6% were up to date with colorectal cancer screening [7]. In a statewide study conducted by our team, 81.3% of Non-Hispanic Blacks were up to date with breast cancer screening, 78.0% were up to date with cervical cancer screening and 78.2% were up to date with colorectal cancer screening. Among Whites living in Indiana, 80.2% were up to date with breast cancer screening, 75.7% were up to date with cervical cancer screening and 80.3% were up to date with colorectal cancer screening [8]. Improving our understanding of the behaviors that place Hispanics at increased risk for, or help to prevent, cancer is a public health priority.

Cancer rates among Hispanics within Indiana and the United States may be attributed to various causes. First, Hispanics are less likely to have health insurance [2, 9]. According to the Indiana Behavioral Risk Factor Surveillance System (BRFSS) in 2020, 32.9% of Hispanics aged 18–64 were uninsured, compared to only 9.0% of non-Hispanic whites [10]. Second, Hispanics are more likely to experience higher rates of poverty. In 2019, 15.3% of Hispanic residents in Indiana were living below the poverty line compared to 10% of non-Hispanic whites [11]. Third, cancer rates among Hispanics in Indiana also may be attributed to high-risk health behaviors. Rates of obesity and physical inactivity among Hispanics are higher in Indiana than the national average [12]. According to the Indiana BRFSS, obesity prevalence in Hispanic men was 32.5%, and 36.6% among women in 2020.[10]. Cigarette smoking rates are lower compared to the rest of the population, both nationally [13] and in Indiana [10] but substantial variation in smoking prevalence exists among Hispanic subgroups [13].

In 2020, Hispanics constituted 8.2% of Indiana’s population with approximately 554,000 residents [5]. However, little is known about their health and their cancer-related health behaviors. To improve health equity for Hispanic residents, research is needed to understand the factors that contribute to cancer risk and risk reduction. Bridging this gap in knowledge will serve Indiana healthcare organizations and providers to better identify the health needs of the state’s Hispanic population.

The objectives of the present study were to: 1) describe cancer beliefs and cancer preventive/risk reduction behaviors (physical activity, tobacco, and alcohol use) among Hispanic adults residing in Indiana; 2) examine differences in cancer beliefs and preventive behaviors by country/territory of birth, socioeconomic status (income, education), and area of residence (urban vs. rural); and 3) determine predictors of engagement in cancer prevention and risk reduction behaviors. The study was approved by a Midwestern university institutional review board (IRB #2002388780) and informed consent was obtained from all participants.

## Methods

Hispanic adults (over age 18) who were currently living in the state of Indiana and could read and write either English or Spanish were recruited using Facebook-targeted advertisement to complete an online survey assessing cancer-related knowledge and beliefs, information-seeking behaviors, health behaviors and cancer prevention strategies. Data were collected through English and Spanish-language online surveys developed in Qualtrics. Upon survey completion, participants received a $15 electronic gift card. We leveraged Qualtrics IP-detection technology to both avoid duplicates and screen out potential respondents outside of our catchment area.

### Measures

To develop the survey, we refined a population health survey that had been used previously with a diverse sample of Indiana residents funded by the National Cancer Institute (P30 CA082709-17S6; Loehrer, PI). Our prior study examined knowledge, beliefs, and behaviors associated with cancer screening and cancer prevention strategies among 980 Indiana adults [8, 14, 15]. Relevant survey items were identified from the Health Information National Trends Survey (HINTS), the Behavioral Risk Factor Surveillance System (BRFSS) survey, and the National Health and Nutrition Examination Survey (NHANES). Survey items assessed the following areas: 1) individual and sociodemographic characteristics, 2) cancer knowledge and beliefs; 3) health promoting/cancer prevention behaviors (diet, physical activity, tobacco use, screening, vaccine); and 4) health information-seeking behaviors and preferences [16].

For the present study, we modified the original survey and translated it to create both English and Spanish versions. A Translation Task Force comprised of five bilingual members of the study team translated, revised, and finalized the Spanish-language survey and study documents (study information sheets, consent forms, etc.). We then conducted cognitive interviews (five in Spanish and five in English) with Hispanic adults residing in Indiana. Cognitive interviews focused on the structure, flow, and layout of the survey, as well as the wording, order, and clarity of questions and images. Input was used to revise and finalize both language versions of the survey. Interviewees received a $50 electronic gift card.

Health behaviors that were measured included human papilloma virus (HPV) vaccination status, engagement in moderate exercise, smoking status, and frequency of alcohol consumption. HPV vaccination was measured with two items: “A vaccine to prevent human papilloma virus or HPV infection is called the cervical cancer or genital warts vaccine, HPV shot GARDASIL or CERVARIX. Have you ever had an HPV vaccination?” The second open-ended item asked, “How many shots did you receive?” Smoking status was measured with a single item “Do you now smoke cigarettes every day, some days, or not at all?” Engagement in moderate exercise was assessed with a single item: “In a typical week, outside of your job or work around the house, how many days do you do any physical activity or exercise of at least moderate intensity such as brisk walking, bicycling at a regular pace, and swimming at a regular pace. Response options ranged from none to 7 days per week. Frequency of alcohol consumption was assessed with a single item: “How often do you have a drink containing alcohol?” Response options were never, monthly or less, 2–4 times per month, 2–3 times per week, 4 or more times per week.

### Recruitment

Our Facebook-targeted advertising recruitment strategy was developed in consultation with an experienced researcher who specialized in social media-based recruitment [17]. First, we created a study page on Facebook that contained a description of the study eligibility criteria, information about how members of the target population could participate, and contact information for study personnel. Next, we used Facebook’s marketing features to distribute two targeted ads – one in English and one in Spanish. The ads included a generic description of the study and a hyperlink to the eligibility screening questionnaire. Eligibility screening questions assessed potential participant’s age, whether they currently resided in Indiana, and whether they identified as Hispanic, Latino, or of Spanish origin. Upon completing the screening questionnaire, eligible participants were directed to the online survey.

### Data Collection

Data collection took place during the COVID-19 pandemic which made the risks of in-person study activities prohibitive. Both the Spanish and English-language online surveys included 78 closed- and 15 open-ended items. The survey took a median of 45.9 min to complete. We designed the survey so that eligible participants could take the survey only once through the “Prevent Ballot Box Stuffing” option in Qualtrics. Participants who completed the survey were sent a $15 Amazon electronic gift card. Prior to distributing this compensation, we manually reviewed each participant’s survey to examine missing data, verify its completion time, and whether the zip code reported was in Indiana.

Two weeks after publishing our Facebook-targeted advertisement, we evaluated the number of completed surveys in each language, and the age, gender, and zip code distributions of participants. We addressed language and age imbalances through pausing the English-language ad and survey and emphasized invitations to target Spanish-speaking participants aged 50 and older. In addition to releasing a revised Facebook ad, we also promoted the study on a local Spanish-language television news broadcast to encourage Spanish-speaking Hispanics in Indiana aged 50 and over to complete the survey. Survey completion rates and limited demographic data were reviewed again two weeks later. To further balance the representation of age groups between the Spanish and English surveys, we adjusted the Spanish-language Facebook ad and Qualtrics survey. After receiving 850 Spanish-language and 791 English-language surveys (1,641 total), we discontinued both Facebook ads and closed both surveys after 26 days.

### Data Analysis

To examine differences in cancer-related beliefs, knowledge, and behaviors of Latino/ Hispanic adults residing in Indiana, we restricted our data to include only surveys that were completed by participants who never had cancer. This filtering criterion resulted in a total number of 1,520 usable surveys returned by Indiana residents across the state. To describe cancer beliefs and cancer prevention and risk reduction behaviors, we calculated frequencies and percentages. We then compared each of these beliefs and behaviors based on country/territory of birth, area of residence (urban vs. rural), income level, and education level using either Chi-square tests or Fisher’s exact tests depending on counts by categories. In multivariate analyses, logistic models were used for binary outcomes (e.g., current smoking = yes/no); logistic multinomial models were used when outcomes were discrete, with more than two possible values (for example “frequency of drinking alcohol” could be never, ≤ 1 time per month, 2–4 times per month, or 2–4 times per week); and linear models were used when outcomes were continuous (days of moderate activity per week, for example). Variables were included in each regression model if they had significant, univariate associations with each model’s outcome variable at a level of *p* ≤ 0.2. All statistical analyses were performed in R v4.0.3 [18] and RStudio v1.2.1335 [19] using base packages and the car package [20]. Urban and rural categories were computed based on the USDA classification of Rural–Urban Commuting Area (RUCA) Codes for zip codes.

## Results

Of the 1,520 surveys, 52% were completed by men with 48% by women. As shown in Table 1, the median age of respondents was 53 years, 70% were White, 46.3% had attended some college or completed college, 76.5% were currently employed, and 87.6% were married or living with a partner. Forty-five percent of respondents owned their own home, 60% were born in the U.S. and 80.9% spoke all or mostly English. While 78% reported an annual household income ranging from $35,000-$74,999, 47% indicated that they were finding it difficult or very difficult to get by on their present income. Eighty percent of respondents resided in an urban area.

**Table 1:** Sample characteristics

Variable		Mean		Standard deviation	Median		*n*
Age		47.26		12.52	53		1520
			*n*			%	
Sex:	Female		726			47.76	
Education:	Less than high school		153			10.07	
	Completed high school		663			43.62	
	Some college		573			37.70	
	Completed college		131			8.62	
Rent or own home:	Rent		534			35.13	
	Own		684			45.00	
	Occupy home with no rent		302			19.87	
Marital status:	Married/Partnered		1332			87.63	
	Not married/Partnered		188			12.37	
Language Spoken with Partner:	All/mostly English		1157			80.91	
	All/mostly Spanish		197			13.78	
	Spanish/English equally		76			5.31	
Race:	White		1073			70.22	
	Black		23			1.51	
	Multiracial		91			5.96	
	Indigenous or Mestizo		169			11.06	
	Other		172			11.26	
Country/Territory of Birth:	Cuba		108			7.11	
	Mexico		208			13.68	
	Puerto Rico		86			5.66	
	USA		918			60.39	
	Other*		200			13.16	
Annual Household Income:	$0 – 34,999		125			8.26	
	$35,000 – 49,999		548			36.22	
	$50,000 – 74,999		635			41.97	
	$75,000 or more		205			13.55	
Financial adequacy:	Finding it difficult/very difficult to get by on present income		717			47.20	
	Getting by on present income		597			39.30	
	Comfortable on present income		205			13.50	
Employment status:	Employed full-time		546			35.92	
	Employed part-time		617			40.59	
	Not currently employed		357			23.49	
Health insurance:	Yes		1265			83.22	
Area of residence:	Urban		1217			80.07	
	Rural		303			19.93	

^*^Other countries included the Dominican Republic, Guatemala, Honduras, El Salvador, Nicaragua, Costa Rica, Columbia, Venezuela, Argentina, Chile, Paraguay, Peru, Bolivia, Ecuador, other, and more than one country

With regards to cancer-related beliefs and cancer prevention behaviors, most respondents (59%) believed they were unlikely to get cancer in their lifetime while 34% were extremely or somewhat worried about getting cancer. Most respondents held fatalistic beliefs about cancer, with 56% agreeing with the statement ‘when I think about cancer, I think about death’. The majority also agreed that ‘it seems like everything causes cancer’ (55%), ‘it’s hard to know what cancer prevention recommendations to follow’ (61%), ‘there’s not much you can do to lower your chances of getting cancer’ (53%), and ‘I’d rather not know about my chances of getting cancer’ (54%). Detailed data about cancer beliefs and cancer screening behaviors were reported earlier [7].

We explored overall rates of cancer prevention/risk reduction behaviors and then examined differences between groups based on their country/territory of birth, area of residence (urban vs. rural), income, and education levels. Overall, 35.6% of eligible participants reported receiving a human papilloma virus (HPV) vaccine and only 72.6% received all three shots. The average days respondents engaged in moderate exercise was 3.55 (s.d = 1.42). Current smoking was reported among 37.6% of our participants and the percentage of respondents who reported never drinking was 11.8%.

When cancer prevention behaviors were stratified by country/territory of birth, significant differences were observed on all but two questions (number of HPV shots and alcoholic drink frequency) (See Table 2). Significant differences were observed by country/territory of birth on HPV vaccination rates (*p* < 0.001), number of days of moderate exercise per week (*p* < 0.001), and smoking rates (*p* = 0.003). Specifically, respondents from the USA, including Puerto Rico, had significantly higher HPV vaccination rates than respondents from Mexico, Cuba, or other Latin American countries. Further, respondents born in Mexico and Cuba reported the highest average days of moderate exercise per week while those from the USA and Mexico had the highest, and equivalent, smoking rates. No significant differences were observed in any of these 5 cancer prevention behaviors by area of residence (Table 2).

**Table 2:** Differences in cancer prevention/risk reduction behaviors by country/territory of birth and area of residence

	Overall *n* (%)	Country/Territory of Birth *n* (%)						Area of Residence *n* (%)		
		USA	Mexico	Cuba	P. Rico	Other	*p*	Urban	Rural	*p*
Received HPV vaccine										
Yes	541 (35.6)	383 (41.8)^abc^	46 (22.1)^ad^	25 (23.1)^be^	34 (39.5)^def^	53 (26.5)^cf^	< 0.001	439 (36.1)	102 (33.7)	0.474
No	979 (64.4)	535 (58.2)	162 (77.9)	83 (76.9)	52 (60.5)	147 (73.5)		778 (63.9)	201 (66.3)	
If yes, number of shots										
1	32 (5.9)	18 (4.7)	7 (15.2)	2 (8)	1 (2.9)	4 (7.5)	0.055	25 (5.7)	7 (6.9)	0.739
2	116 (21.4)	92 (24.0)	2 (4.3)	3 (12)	4 (11.8)	15 (28.3)		92 (20.9)	24 (23.5)	
3	393 (72.6)	273 (71.3)	37 (80.4)	20 (80)	29 (85.3)	34 (64.2)		322 (73.3)	71 (69.6)	
Days of moderate exercise per week										
Mean (SD)	3.55 (1.42)	3.41 (1.44)^ab^	4.04 (1.30)^ac^	4.04 (1.20)^bd^	3.71 (1.37)^e^	3.30 (1.41)^cde^	< 0.001	3.52 (1.44)	3.65 (1.35)	0.139
Current smoker										
Yes	572 (37.6)	371 (40.4)^ab^	84 (40.4)^cd^	38 (35.2)	23 (26.7)^ac^	56 (28.0)^bd^	0.003	469 (38.5)	103 (34.0)	0.163
No	948 (62.4)	547 (59.6)	124 (59.6)	70 (64.8)	63 (73.3)	144 (72.0)		748 (61.5)	200 (66.0)	
Alcoholic drink frequency										
Never	179 (11.8)	106 (11.5)	24 (11.5)	12 (11.1)	9 (10.5)	28 (14.0)	0.219	141 (11.6)	38 (12.5)	0.822
> Once a month	369 (24.3)	229 (24.9)	51 (24.5)	20 (18.5)	19 (22.1)	50 (25.0)		292 (24.0)	77 (25.4)	
2–4 times/mo	671 (44.1)	401 (43.7)	82 (39.4)	55 (50.9)	39 (45.3)	94 (47.0)		538 (44.2)	133 (43.9)	
2 + times/week	301 (19.8)	182 (19.8)	51 (24.5)	21 (19.4)	19 (22.1)	28 (14.0)		246 (20.2)	55 (18.2)	

Matching superscript letters denote significant pairwise comparisons, at the < 0.05 level, by row. E.g. USA and Mexico have significantly different average days of exercise, so both are denoted with “a”. Within that same row (days of moderate exercise per week), USA and Cuba have superscript “b” so they are significantly different, Mexico and “Other” have superscript “c” so they are significantly different, as are Cuba and “Other” with superscript “d”

Several significant differences were observed in behaviors by levels of income and education (see Table 3). Generally, significant differences were found across income levels in the number of HPV shots received (*p* = 0.009), the number who currently smoke cigarettes (*p* = 0.018), the frequency of drinking alcohol (*p* < 0.001), and the number of days engaging in moderate physical activity per week (*p* < 0.001). Individuals in the lowest income group were found to drink and smoke significantly less than those with higher incomes. Conversely, respondents with low incomes were more likely to report they received the full course of 3 HPV shots than individuals with higher incomes. Similar differences were observed across education levels.

**Table 3:** Differences in cancer prevention/risk reduction behaviors by income and education

	Overall *n* (%)	Income *n* (%)					Education *n* (%)				
		0 to $34,999	$35,000 to $49,999	$50,000 to $74,999	$75,000 +	*P*	Less than high school	Completed high school	Some college	College degree or higher	*P*
Received HPV vaccine											
Yes	541 (35.6)	47 (37.6)	207 (37.8)	229 (36.1)	56 (27.3)	0.057	49 (32.0)	227 (34.2)	222 (38.7)	43 (32.8)	0.231
No	979 (64.4)	78 (62.4)	341 (62.2)	406 (63.9)	149 (72.7)		104 (68.0)	436 (65.8)	351 (61.3)	88 (67.2)	
If yes, number of shots											
1	32 (5.9)	4 (8.5)^abc^	11 (5.3)^a^	14 (6.1)^b^	3 (5.4)^c^	0.009	1 (2.0)^a^	10 (4.4)^b^	17 (7.7)^c^	4 (9.3)^abc^	0.003
2	116 (21.4)	1 (2.1)	39 (18.8)	57 (24.9)	18 (32.1)		9 (18.4)	38 (16.7)	51 (23.0)	18 (41.9)	
3	393 (72.6)	42 (89.4)	157 (75.8)	158 (69.0)	35 (62.5)		39 (79.6)	179 (78.9)	154 (69.3)	21 (48.8)	
Days of moderate exercise per week											
Mean (SD)	3.55 (1.42)	3.39 (1.54)^a^	3.75 (1.34)^ab^	3.41 (1.46)^b^	3.54 (1.34)	< 0.001	3.79 (1.31)^ab^	3.82 (1.26)^cd^	3.35 (1.50)^ace^	2.76 (1.50)^bde^	< 0.001
Current smoker											
Yes	572 (37.6)	31 (24.8)^abc^	213 (38.9)^a^	251 (39.5)^b^	76 (37.1)^c^	0.018	43 (28.1)^ab^	275 (41.5)^ac^	221 (38.6)^bd^	33 (25.2)^cd^	< 0.001
No	948 (62.4)	94 (75.2)	335 (61.1)	384 (60.5)	129 (62.9)		110 (71.9)	388 (58.5)	352 (61.4)	98 (74.8)	
Alcoholic drink frequency											
Never	179 (11.8)	21 (16.8)^abc^	71 (13.0)^a^	61 (9.6)^b^	24 (11.7)^c^	< 0.001	26 (17.0)^a^	51 (7.7)^abc^	80 (14.0)^b^	22(16.8)^c^	< 0.001
< once per month	369 (24.3)	56 (44.8)	130 (23.7)	136 (21.4)	43 (21.0)		29 (19.0)	179 (27.0)	125 (21.8)	36 (27.5)	
2–4 × per month	671 (44.1)	33 (26.4)	228 (41.6)	306 (48.2)	103 (50.2)		70 (45.7)	287 (43.3)	262 (45.7)	52 (39.7)	
> 2 × per week	301 (19.8)	15 (12.0)	119 (21.7)	132 (20.8)	35 (17.1)		28 (18.3)	146 (22.0)	106 (18.5)	21 (16.0)	

Matching superscript letters denote significant pairwise comparisons, at the 0.05 level, by row

When we examined pairwise comparisons in these measures across education levels the patterns were more complex. Participants who had completed college or higher had received the fewest number of HPV shots, and exercised the fewest number of days on average. Those who completed high school or had some college had the highest percentage of people who smoked cigarettes. Finally, the frequency of drinking alcohol was similar across education levels except for those who completed high school; this group had the lowest percentage who never drank alcohol (see Table 3).

To clarify these relationships, we examined predictors cancer prevention/risk reduction behaviors using multivariate regression models. Our first model examined the probability of participants having received the HPV vaccine based on a variety of sociodemographic, and cancer belief variables. From this model, we saw that individuals who were less worried about getting cancer (*p* < 0.001), males (*p* < 0.001), higher income earners (*p* = 0.018), those born outside of the USA (*p* < 0.001), and those employed less than full-time (*p* < 0.001) were less likely to have received the HPV vaccine. Conversely, we saw that individuals who reported higher levels of financial adequacy (*p* < 0.001), and white individuals (*p* = 0.022) were more likely to have received the HPV vaccine (Table 4).

**Table 4:** Multivariate logistic regression model predicting HPV vaccination status

		HPV Vaccination Status	
*Predictors*		*Odds Ratio (CI)*	*p*
How worried are you about getting cancer?			
	Extremely/Moderately (*n* = 511)	Ref	** < 0.001**
	Somewhat (*n* = 549)	0.63 (0.48 – 0.83)^**^	
	Slightly/Not at all (*n* = 449)	0.53 (0.39 – 0.72)^***^	
Financial Adequacy	Finding it difficult/very difficult on present income (*n* = 714)	Ref	** < 0.001**
	Getting by on present income (*n* = 591)	1.75 (1.34 – 2.29)^***^	
	Living comfortably on present income (*n* = 204)	1.82 (1.24 – 2.66)^***^	
Gender	Female (*n* = 719)	Ref	** < 0.001**
	Male (*n* = 789)	0.58 (0.46 – 0.72)^***^	
Annual Household Income	$0 to $34,999 (*n* = 124)	Ref	**0.018**
	$35,000 to $49,999 (*n* = 547)	0.91 (0.59 – 1.41)	
	$50,000 to $74,999 (*n* = 634)	0.73 (0.47 – 1.13)	
	$75,000 + (*n* = 204)	0.51 (0.30 – 0.87)^*^	
Country/Territory of Birth	USA (*n* = 915)	Ref	** < 0.001**
	Mexico (*n* = 206)	0.51 (0.30 – 0.87)^***^	
	Cuba (*n* = 107)	0.57 (0.34 – 0.94)^*^	
	Puerto Rico (*n* = 85)	1.20 (0.73 – 1.96)	
	Other (*n* = 196)	0.52 (0.35 – 0.75)^***^	
Employment status	Employed full-time (*n* = 541)	Ref	** < 0.001**
	Employed part-time (*n* = 615)	0.59 (0.45 – 0.78)^***^	
	Not currently employed (*n* = 353)	0.55 (0.39 – 0.76)^***^	
Race	Non-white (*n* = 446)	Ref	**0.022**
	White (*n* = 1063)	1.31 (1.00 – 1.72)^*^	

Odds ratios, and 95% CI of model on HPV vaccine status (*n* = 1509), *R*^*2*^ = 0.108 * *p* < 0.05 ** *p* < 0.01 *** *p* < 0.001

This table includes just the factors found to be significant from an ANOVA F-test at the level of *p* ≤ 0.05. Other, non-significant variables entered into this model were: age, “how likely are you to get cancer in your life?”, education, financial adequacy, income ranges, marital/partnered status, race, “rent or own home?”, “it’s hard to know which cancer prevention recommendations to follow”, and area of residence

Table 5 shows our multivariate logistic regression model predicting current smoking status where males were 9.5 times more likely to currently smoke than females (*p* < 0.001), and part-time workers and unemployed individuals also were more likely to smoke cigarettes than full-time workers (*p* = 0.038). Conversely, individuals who were not exclusively English speakers were significantly less likely to currently smoke (*p* = 0.002) as were individuals who were born in Puerto Rico and South and Central American countries (*p* = 0.016).

**Table 5:** Multivariate logistic regression model predicting smoking status

		Currently smoke	
*Predictors*		*Odds Ratios (CI)*	*p*
Language Spoken with Partner	All or Mostly English (*n* = 1153)	Ref	**0.002**
	All or Mostly Spanish (*n* = 194)	0.59 (0.49 – 0.89)^*^	
	Spanish & English equally (*n* = 73)	0.41 (0.21 – 0.77)^**^	
Country/Territory of Birth	USA (*n* = 856)	Ref	**0.016**
	Mexico (*n* = 194)	1.16 (0.77 – 1.74)	
	Cuba (*n* = 106)	0.66 (0.40 – 1.09)	
	Puerto Rico (*n* = 82)	0.51 (0.28 – 0.91)^*^	
	Other (*n* = 182)	0.64 (0.42 – 0.98)^*^	
Employment status	Employed full-time (*n* = 487)	Ref	**0.038**
	Employed part-time (*n* = 600)	1.46 (1.08 – 2.00)^*^	
	Not currently employed (*n* = 333)	1.45 (1.00 – 2.12)	

Odds ratios, and 95% CI of model on current smoking status (*n* = 1420), *R*^*2*^ = 0.257; * *p* < 0.05 ** *p* < 0.01 *** *p* < 0.001

This table includes just the factors found to be significant from an ANOVA F-test at the level of *p* ≤ 0.05. Other, non-significant variables entered into this model were: age, “how likely are you to get cancer in your life?”, education, financial adequacy, income ranges, marital/partnered status, race, “rent or own home?”, “it’s hard to know which cancer prevention recommendations to follow”, area of residence, and health insurance status

We then constructed a linear regression model to examine predictors of the number of days of moderate physical activity per week, a continuous outcome (Table 6). From this model we saw that the average number of days of physical activity per week across the entire sample was 2.07 and that individuals who were less worried about getting cancer or assessed their risk of getting cancer as unlikely reported more days of moderate physical activity (*p* < 0.001). Similarly, we saw that males (*p* < 0.001), higher income earners (*p* < 0.001), individuals from Mexico (*p* = 0.02), and those not employed or employed less than full-time (*p* < 0.001) had more days of moderate physical activity per week. Interestingly, while higher income levels were associated with more days of physical activity generally, greater financial adequacy predicted a lower number of days of physical activity per week (*p* < 0.001). This suggested that the financial adequacy variable is capturing some variation not directly related to household income (Table 6).

**Table 6:** Multivariate logistic regression model predicting days of moderate physical activity

		Days of moderate activity/week	
*Predictors*		*Estimates (CI)*	*p*
(Intercept)		2.07 (1.56 – 2.59)^***^	
How worried are you about getting cancer?			
	Extremely/Moderately (*n* = 468)	Ref	** < 0.001**
	Somewhat (*n* = 524)	0.47 (0.33 – 0.62)^***^	
	Slightly/Not at all (*n* = 426)	0.57 (0.41 – 0.74)^***^	
How likely are you to get cancer in your lifetime?	Slightly/Not at all (*n* = 426)	Ref	** < 0.001**
	Neither likely nor unlikely (*n* = 150)	0.01 (-0.20 – 0.22)	
	Unlikely (*n* = 867)	0.84 (0.68 – 0.99)^***^	
Financial adequacy	Finding it difficult/very difficult on present income (*n* = 699)	Ref	** < 0.001**
	Getting by on present income (*n* = 540)	-0.46 (-0.60 – -0.32)^***^	
	Living comfortably on present income (*n* = 179)	-0.76 (-0.97 – -0.55)^***^	
Gender	Female (*n* = 670)	Ref	** < 0.001**
	Male (*n* = 747)	0.22 (0.10 – 0.35)^***^	
Annual Household Income	$0 to $34,999 (*n* = 111)	Ref	** < 0.001**
	$35,000 to $49,999 (*n* = 520)	0.43 (0.20 – 0.67)^***^	
	$50,000 to $74,999 (*n* = 593)	0.46 (0.22 – 0.70)^***^	
	$75,000 + (*n* = 179)	0.68 (0.41 – 0.96)^***^	
Country/Territory of Birth	USA (*n* = 855)	Ref	**0.02**
	Mexico (*n* = 193)	0.31 (0.12 – 0.49)^**^	
	Cuba (*n* = 106)	0.14 (-0.10 – 0.38)	
	Puerto Rico (*n* = 82)	-0.07 (-0.33 – 0.19)	
	Other (*n* = 182)	0.08 (-0.11 – 0.28)	
Employment status	Other (*n* = 182)	Ref	** < 0.001**
	Employed part-time (*n* = 599)	0.53 (0.39 – 0.68)^***^	
	Not currently employed (*n* = 332)	0.38 (0.21 – 0.55)^***^	

Beta coefficients and 95% CI of model on physical activity (*n* = 1418), *R*^*2*^ = 0.376

^*^ *p* < 0.05 ** *p* < 0.01 *** *p* < 0.001

This table includes just the factors found to be significant from an ANOVA F-test at the level of *p* ≤ 0.05. Other, non-significant variables entered into this model were: age, “how likely are you to get cancer in your life?”, education, financial adequacy, income ranges, marital/partnered status, race, “rent or own home?”, “it’s hard to know which cancer prevention recommendations to follow”, area of residence, and health insurance status

Finally, to examine predictors of frequency of alcohol consumption, we constructed a multivariate, multinomial logistic regression model (Table 7). Frequency of alcohol consumption had 4 response options ranging from: never drink alcohol (never) to drink ≤ once per month (rarely), 2–4 times per month (occasionally), or ≥ 2 times per week (frequently). In addition to the other significant effects reported in this table, we want to highlight that this model showed that, for every year of increasing age, participants were more likely to drink rarely as compared to never drinking (*p* = 0.003), the reference level for the outcome. Those who completed high school were more likely to drink both occasionally and frequently compared to individuals who did not complete high school, while participants who completed college were more likely to drink occasionally compared to those who did not complete high school (*p* = 0.015).

**Table 7:** Multivariate, multinomial logistic regression model predicting frequency of drinking alcohol

	Never		≤ 1 time/month (Rarely)		2–4 times/month (Occasionally)		> 2 times/week (Frequently)		
*Predictors*	*Odds Ratio*	*n*	*Odds Ratio (CI)*	*n*	*Odds Ratio (CI)*	*n*	*Odds Ratio (CI)*	*n*	*p*
Age (*n* = 1420)	Ref		1.04 ^**^ (1.01,1.06)		1.01 (0.99,1.03)		1.01 (0.99, 1.03)		**0.003**
How worried are you about getting cancer?									
Extremely/Moderately	ref	58	ref	92	ref	228	ref	90	**0.028**
Somewhat	ref	49	1.69 (1.00, 2.87)	130	1.57 (0.97, 2.54)	247	1.18 (0.68, 2.06)	98	
Slightly/Not at all	ref	50	1.23 (0.70, 2.14)	116	0.81 (0.48, 1.35)	159	0.95 (0.53, 1.71)	103	
How likely are you to get cancer in your lifetime?									
Likely	ref	52	ref	66	ref	216	ref	67	**0.012**
Neither likely nor unlikely	ref	20	2.47^**^ (1.25, 4.90)	48	1.05 (0.56, 1.97)	62	1.07 (0.49, 2.32)	21	
Unlikely	ref	85	1.51 (0.89, 2.58)	224	0.93 (0.58, 1.50)	356	1.25 (0.71, 2.18)	203	
Education									
Less than high school	ref	22	ref	28	ref	65	ref	27	**0.015**
Completed high school	ref	44	2.95^**^ (1.47, 5.91)	166	1.66 (0.89, 3.09)	278	2.18^*^ (1.06, 4.51)	144	
Some college or university	ref	73	1.68 (0.81, 3.49)	115	0.93 (0.48, 1.78)	248	1.12 (0.52, 2.43)	100	
Completed college or higher	ref	18	2.78^*^ (1.09, 7.09)	29	0.99 (0.43, 2.31)	43	2.31 (0.84, 6.36)	20	
It seems like everything causes cancer									
Agree	ref	106	ref	180	ref	341	ref	145	**0.025**
Disagree	ref	51	1.82^**^ (1.16, 2.86)	158	1.88^**^ (1.24, 2.86)	293	1.78^*^ (1.11, 2.85)	146	
It’s hard to know which cancer prevention recommendations to follow									
Agree	ref	99	ref	184	ref	369	ref	192	**0.022**
Disagree	ref	58	1.45 (0.93, 2.25)	154	1.41 (0.94, 2.11)	265	0.90 (0.56, 1.44)	99	
Financial Adequacy									
Finding it difficult/very difficult on present income	ref	66	ref	175	ref	287	ref	171	**0.017**
Getting by on present income	ref	63	0.99 (0.61, 1.61)	134	0.96 (0.61, 1.51)	250	0.60 (0.36, 1.00)	95	
Living comfortably on present income	ref	28	0.56 (0.28, 1.15)	29	0.89 (0.48, 1.65)	97	0.39^*^ (0.19, 0.83)	25	
Gender									
Female	ref	109	ref	192	ref	306	ref		** < 0.001**
Male	ref	47	1.58^*^ (1.02, 2.45)	146	2.40^***^ (1.60,3.61)	328	8.32^***^ (5.18,13.34)		
Annual Household Income									
$0 to $34,999	ref	17	ref	51	ref	31	ref	13	** < 0.001**
$35,000 to $49,999	ref	66	0.40^*^ (0.19, 0.83)	118	1.10 (0.52, 2.31)	219	1.65 (0.68, 4.04)	117	
$50,000 to $74,999	ref	52	0.61 (0.29, 1.30)	128	1.80 (0.85, 3.84)	286	2.65^*^ (1.07, 6.56)	127	
$75,000 +	ref	22	0.36^*^ (0.15, 0.87)	41	1.24 (0.52, 2.93)	98	1.55 (0.55, 4.37)	34	
Language Spoken with Partner									
All/Mostly English	ref	102	ref	284	ref	519	ref	248	**0.030**
All/Mostly Spanish	ref	43	0.38^**^ (0.21, 0.68)	34	0.47^**^(0.28, 0.78)	82	0.46^*^ (0.25, 0.84)	35	
Spanish & English equally	ref	12	0.76 (0.32, 1.79)	20	0.64 (0.29, 1.41)	33	0.41 (0.15, 1.14)	8	
Race									
Non-white	ref	44	ref	84	ref	212	ref	64	**0.019**
White	ref	113	0.94 (0.58, 1.53)	254	0.68 (0.44, 1.05)	422	1.10 (0.66, 1.85)	227	

Odds ratios, and 95% CI of model on alcoholic drink frequency (*n* = 1420), *R*^*2*^ = 0.390; * *p* < 0.05 ** *p* < 0.01 *** *p* < 0.001

This table includes just the factors found to be significant from an ANOVA F-test at the level of *p* ≤ 0.05. Other, non-significant variables entered into this model were: “When I think about cancer, I think about death”, “Correctly identified age from Lung Cancer screening guidelines”, Marital/partnered status, Occupation status, “You can’t lower your cancer risk much”, “Rent or own home?”, and, and health insurance status. Also note that the scale for this response ranges from never having alcoholic drinks, to having alcoholic drinks 5 + times per month. This last category includes people who answered that they have alcoholic drinks up to 4 + times per week

Men were more likely than women to drink rarely, occasionally, and frequently rather than never (*p* < 0.001). Finally, there was a strong effect of household income (*p* < 0.001) where individuals earning $35,000 to $49,999 and $75,000 + per year were less likely than individuals earning < $35,000 per year to drink rarely as opposed to never drink. Conversely, participants who earned $50,000 to $74,999 per year were 2.6 times more likely than those with lower incomes to drink frequently (≥ 2 times per week) as opposed to never drinking. Regarding associations between alcohol consumption and health beliefs, participants who rated their chance of getting cancer as “neither likely nor unlikely” compared to those who thought they were “likely” to get cancer were more likely to drink rarely, as opposed to never drinking. Participants who disagreed with the statement that “everything causes cancer” were more likely to drink rarely, occasionally, and frequently rather than never drink compared to those who agreed that “everything causes cancer” (*p* = 0.025).

## Discussion

Nationally, Hispanics comprise the largest and youngest non-white community in the U.S. with most of this population residing in the Southwest and Florida. Hispanics are the most rapidly growing population in the U.S. and approximately two-thirds are born in the U.S. [9]. This population is incredibly diverse with people of many races, religions, languages, and cultural identities. The majority of U.S. Hispanics identify as Mexican (61.9%), followed by Puerto Rican, (9.7%), Cuban (4.0%) Salvadoran (3.9%) and Dominican (3.4%) but the distribution varies greatly by state [9]. In this study, conducted in Indiana, our distribution was similar to national data; 60% of our participants were born in the U.S. followed by 13.7% born in Mexico, 7.1% in Cuba, and 5.7% in Puerto Rico.

Socioeconomic factors, specifically income and education, are strongly associated with health, longevity, and quality of life [6, 9]. Studies have shown that Hispanics living in the U.S. have lower levels of education and are more likely to live in poverty compared to non-Hispanic whites, but large differences by country/territory of birth exist [9]. Our findings were consistent with other studies showing Hispanics have lower levels of education; only 46% of our Hispanic sample had attended or completed college compared to 65% of Indiana residents who were non-Hispanic white [5]. However, differences in annual household incomes between Hispanic and non-Hispanic whites in Indiana were very small; 44.5% of our Hispanic respondents in this study reported earning less than $50,000 annually compared to 46.6% of non-Hispanic whites residing in Indiana [8].

Cancer beliefs reflect people’s concerns about getting cancer, their perception of their own susceptibility to the disease, its causes and consequences, and influence their willingness to engage in cancer risk-reduction behaviors. A surprising majority (59%) of our participants reported they were unlikely or very unlikely to get cancer in their lifetime. This proportion was much higher than non-Hispanic whites (14.1%) and Blacks (21.6%) in Indiana who believed they were unlikely to get cancer [21]. Given the generally low cancer risk in this population, this may reflect a somewhat accurate perception. However, acculturation in the U.S. significantly increases cancer risk. Cancer death rates were 60% higher among Hispanic men who were born in the U.S. compared to those born outside the U.S. [22]. Death rates among Hispanics approach or surpass those of non-Hispanic whites in the U.S. [4]. Since Hispanic population growth is now fueled by births rather than immigration, the cancer burden among Hispanics, both incidence and mortality rates, will continue to grow [4]. Compared to our prior study comprised primarily of non-Hispanic Indiana residents, greater proportions of our respondents in this study agreed that ‘there is not much you can do to lower your chances of getting cancer (53%)’ and ‘I’d rather not know my chances of getting cancer’ (54%). These findings were consistent with those of Davis and colleagues [23].

Cancer behaviors differed by country/territory of birth, income, and education but not by area of residence (rural vs. urban), although the number of rural residents was relatively small (19.9%). Behaviors that differed significantly by country/territory of birth included receipt of the HPV vaccine, days of moderate exercise per week, and current smoking rates. HPV vaccination rates were highest among those born in the US and Puerto Rico. Days of moderate exercise were highest among those born in Mexico and Cuba which may reflect the nature of the physical types of work done by these residents. Smoking rates are similar and highest among those born in the US and Mexico indicating the need for smoking cessation programs and interventions among these Hispanic subgroups.

Several behaviors differed by education and income levels including days of moderate exercise, cigarette smoking, and frequency of alcohol drinks consumed. Smoking rates among our sample of Hispanics in Indiana was 37.6% and consistent with state-level data showing 36% of Indiana residents reported smoking on every day or on some days in 2019 [10]. This finding, however, is inconsistent with national data as was our finding that the smoking rate was lowest among those with the lowest income. However, smoking rates were lowest among those with a college degree or higher which is consistent with national data. Consumption of alcohol was higher among Hispanics in Indiana compared to the Indiana population as a whole. The percentage of respondents in our study who reported never drinking was 11.8% compared to 51.1% of all Indiana residents. Alcohol consumption was highest among those who earned from $35–49,999 and those who completed high school. Numerous predictors of cancer-related behaviors, including receipt of HPV vaccination, days of moderate physical activity, cigarette smoking, and consumption of alcohol were identified. These predictors included cancer beliefs, demographic variables, language preference, and country/territory of birth which can provide important guidance for future interventions.

Results of this study should be interpreted considering several limitations. The cross-sectional study design does not allow for inferences about causality. The voluntary nature of online recruitment methods means that the sample may not be representative of Latinos residing in Indiana, although comparisons between this state-based sample and U.S. Hispanic populations showed similar patterns in terms of country/territory of birth and education. Hispanics living in Indiana may not be representative of Hispanics residing in other parts of the U.S as immigrations pattern vary by state and region; however, Latinos from Midwestern regions are under-represented in the public health literature regarding patterns of cancer preventive behaviors. While the survey questions used in this study were adapted from a prior study, including well-validated, commonly used items from large national surveys, and were translated using robust translation practices, they have not been validated in Spanish-speaking individuals. Strengths of this study include the large sample size, the diverse representation of participants who were born in and outside of the USA, and the bilingual nature of the participants.

This study has practice implications for developers of health promotion and cancer risk reduction programs in Hispanic communities. Given several important differences in the Hispanic population by country/territory of birth, effective programs/interventions for cancer prevention/risk reduction need to be culturally targeted based on country/territory of birth and individually tailored based on cancer-related beliefs. [23]. Evidence-based interventions for reducing the cancer burden among Hispanics include the use of community-based programs, lay health advisors, and navigators. [24–26]. Awareness of personal cancer risk, risk factors, and steps that can be taken to reduce cancer risk are low in this population, so programs should include information to increase awareness/knowledge of these factors and assistance with behavior change. Efforts to increase receipt of HPV vaccinations in eligible populations need to target health care providers, parents of adolescents, and adults. The Centers for Disease Control and Prevention (CDC) recommends that males and females aged 11–12 years should be vaccinated as well as people aged 9–26 years. Adults aged 27 to 45 years who have not been vaccinated may decide to get the vaccine after talking with their doctors about their risks for new HPV infections and benefits of vaccination for them (https://www.cdc.gov/vaccines/vpd/hpv/hcp/recommendations.html). Resources for parents and providers are readily available from the CDC at https://www.cdc.gov/hpv/parents/vaccine-for-hpv.html; https://www.cdc.gov/hpv/hcp/index.html. Approximately 42% of cancer cases and 45% of cancer deaths are linked to modifiable risk factors and could be prevented if people changed their behaviors, especially cigarette smoking, alcohol consumption, physical inactivity, and HPV vaccinations. One of our most urgent priorities in Indiana must be to provide access to culturally targeted and tailored smoking cessation programs for Hispanic residents.

## Data Availability

Deidentified data from this study are available upon request from the corresponding author.
